# Supplementary material for: Development of a computer-aided design software for the quantitative evaluation of aesthetic damage
Source: PLoS One. 2019 Dec 18;14(12):e0226322. doi: 10.1371/journal.pone.0226322 (PMC6919621; doi:10.1371/journal.pone.0226322)
Supplement: S1 Code site — 1. (ZIP) [file pone.0226322.s001.zip › [EESC_jr][Projeto]Relatorio_07_21/reports/templates_zip/startbootstrap-landing-page-gh-pages.zip!/startbootstrap-landing-page-gh-pages/index.html]

Landing Page - Start Bootstrap Theme


Toggle navigation

Start Bootstrap

- About
- Services
- Contact


# Landing Page

### A Template by Start Bootstrap

---

- Twitter
- Github
- Linkedin


---

## Death to the Stock Photo: Special Thanks

A special thanks to Death to the Stock Photo for providing the photographs that you see in this template. Visit their website to become a member.

---

## 3D Device Mockups by PSDCovers

Turn your 2D designs into high quality, 3D product shots in seconds using free Photoshop actions by PSDCovers! Visit their website to download some of their awesome, free photoshop actions!

---

## Google Web Fonts and Font Awesome Icons

This template features the 'Lato' font, part of the Google Web Font library, as well as icons from Font Awesome.


## Connect to Start Bootstrap:

- Twitter
- Github
- Linkedin


- Home
- ⋅
- About
- ⋅
- Services
- ⋅
- Contact

Copyright © Your Company 2014. All Rights Reserved
